# Impact of Alkali-Activated Tannery Sludge-Derived Geopolymer Gel on Cement Properties: Workability, Hydration Process, and Compressive Strength

**DOI:** 10.3390/gels11050339

**Published:** 2025-05-01

**Authors:** Shoukai Chen, Beiying Liu, Phu Minh Vuong Nguyen, Jinping Liu, Jialin Chen, Fei Zhou

**Affiliations:** 1 School of Water Conservaney, North China University of Water Resources and Electric Power, Zhengzhou 450046, China; man200177@163.com (S.C.); suo20202020@outlook.com (B.L.); liujp@radi.ac.cn (J.L.); 2 Central Mining Institute, National Research Institute, 40-166 Katowice, Poland; pnguyen@gig.eu; 3 School of Mechanics & Civil Engineering, China University of Mining and Technology, Xuzhou 221116, China; feizhou@cumt.edu.cn

**Keywords:** tannery sludge, alkali activation, geopolymer gel, cement

## Abstract

The utilization of tannery sludge (TS) in construction materials not only effectively reduces pollution and resource consumption associated with waste disposal, but also promotes low carbon transformation in the building materials sector, further advancing sustainable development of green construction. This study aims to investigate the impact of sludge-based geopolymer gel on cementitious material performance, revealing the evolution mechanisms of material fluidity, setting time, hydration process, and compressive strength under the coupled effects of tannery sludge and alkali activation, thereby providing a reusable technical pathway to address the resource utilization challenges of similar special solid wastes. A series of alkali-activated composite cementitious materials (AACC) were prepared in the study by partially substituting cement with alkaline activators, TS, and fly ash (FA), through adjustments in TS–FA ratios and alkali equivalent (AE) variations. The workability, hydration process, and compressive strength evolution of AACC were systematically investigated. The experimental results indicated that as the TS content increased from 0% to 100%, the fluidity of fresh AACC decreased from 147 mm to 87 mm, while the initial and final setting times exhibited an exponential upward trend. The incorporation of TS was found to inhibit cement hydration, though this adverse effect could be mitigated by alkaline activation. Notably, 20–40% sludge dosages (SD) enhanced early-age compressive strength. Specifically, the compressive strength of the 0% TS group at 3 d age was 24.3 MPa, that of the 20% TS group was 25.9 MPa (an increase rate of 6.58%), and that of the 40% TS group was 24.5 MPa (an increase rate of 0.82%), whereas excessive additions resulted in the reduction of hydration products content and diminished later stage strength development. Furthermore, the investigation into AE effects revealed that maximum compressive strength (37.4 MPa) was achieved at 9% AE. These findings provide critical data support for realizing effective utilization of industrial solid wastes.

## 1. Introduction

The tanning industry, as a crucial global light industrial sector, generates substantial quantities of highly polluting solid waste tannery sludge (TS), while fulfilling human demand for leather products [1,2,3]. TS primarily originates from wastewater treatment processes associated with leather manufacturing stages including tanning, dyeing, degreasing, and other processing operations. This byproduct has complex compositional characteristics, containing elevated concentrations of organic matter, heavy metals, sulfides, and high salinity levels as principal contaminants [4,5]. Global statistics indicate that the tanning industry generates approximately 5.4 million metric tons of solid liquid composite waste annually, with only 20–25% of raw hides ultimately converted into leather products while the remainder becomes processing waste requiring disposal [6]. Confronted with this substantial sludge output, conventional treatment approaches face significant challenges: landfilling becomes constrained due to risks of heavy metal leaching and excessive land resource consumption, while incineration, although achieving volume reduction, substantially increases flue gas treatment costs due to high temperature volatilization of heavy metals and dioxin formation [7,8,9]. In recent years, researchers have attempted to achieve green treatment and resource utilization of chrome TS through construction material-oriented approaches such as producing ceramics, bricks, or cement admixtures [10,11,12,13,14]. Although these methods demonstrate promising prospects, the costs associated with pollution control during the manufacturing process and the long-term risk of heavy metal leaching [15] continue to hinder large scale application of TS in construction materials.

As a green cementitious material with substantial developmental prospects, alkali-activated cementitious materials employ industrial byproducts such as fly ash (FA) and slag, or other silicon aluminum oxygen rich aluminosilicate precursors. Through the introduction of alkaline activators as chemical stimulants, these materials achieve accelerated reaction kinetics and hardening processes. They exhibit not only superior mechanical properties and favorable workability but also demonstrate corrosion resistance and fire resistant characteristics [16,17,18,19,20]. Compared with conventional Portland cement, alkali-activated cementitious materials reduce carbon emissions by 60 to 80%, primarily attributable to their utilization of reactive aluminosilicate components as binding matrices, thereby eliminating energy intensive processes like high temperature limestone calcination [21,22,23]. Of particular significance, the alkali-activated system demonstrates unique advantages in heavy metal immobilization [24,25,26] as follows: (1) alkaline activators facilitate the formation of a dense three dimensional network structure through dissolution polycondensation reactions of silicon aluminum components from precursors, effectively encapsulating heavy metal ions; (2) active species interact with heavy metal ions through coordination bonding to generate stable zeolite type phases; and (3) the highly alkaline environment promotes the physical encapsulation of heavy metals in C-S-H gel and reduces their mobility. This distinctive advantage establishes a viable technical pathway for TS valorization.

Nevertheless, the elevated production costs of alkaline activators render the alkali-activated TS cementitious system theoretically feasible but economically unviable for engineering applications. To address this limitation, researchers have developed hybrid alkali-activated cement (HAAC) systems through strategic integration of alkali-activated materials with conventional Portland cement, achieving an optimal balance between economic feasibility and environmental performance. For instance, A.S. Palomo et al. [27] formulated HAAC using cement, industrial aluminosilicate glass waste, and alkaline activators, with experimental testing demonstrating superior early age strength development and carbon reduction effectiveness. Zhu et al. [28] engineered HAAC incorporating granulated blast furnace slag, ordinary Portland cement, and alkaline activators, revealing through comparative analysis that HAAC systems exhibit enhanced mechanical strength and reduced overall costs compared to pure alkali-activated counterparts. Sungwoo Park et al. [29] developed HAAC composites from cement, metakaolin, and alkaline activators, identifying through microstructural characterization that HAAC specimens possess more homogeneous and compact microstructures compared to standalone geopolymers and Portland cement groups, fundamentally explaining their superior compressive strength. As an effective approach for the resource utilization of tannery sludge, the incorporation of tannery sludge into HAAC has gradually attracted researchers’ attention. For example, Bie et al. [15] prepared and tested the acid and alkali resistance of alkali-activated composite cement-based cementitious materials, finding that its compressive strength increased with the rise in solution pH value. Jin et al. [30] employed tannery sludge and metakaolin to prepare alkali-activated composite cement-based cementitious materials, successfully shortening its setting time to 102 min and achieving early-age mechanical properties with a 1-day compressive strength reaching 21 MPa. Chen et al. [31] prepared alkali-activated composite cement-based cementitious materials using blast furnace slag and tannery sludge as raw materials and found that its 28-day strength could reach 71.3 MPa. The aforementioned studies provided strong references for the feasibility of tannery sludge as geopolymer raw materials. However, this literature focused more on the macroscopic mechanical characterization of alkali-activated composite cement-based cementitious materials after compounding with aggregates, while lacking studies on the effects of alkali-activation and tannery sludge content variations on alkali-activated composite cement-based cementitious materials performance parameters.

In the composite cementitious system composed of tannery sludge, industrial waste residues, cement, and alkali activators, the interactive effect between sludge content and alkali equivalent has a significant regulatory role on alkali-activated composite cement-based cementitious materials performance. However, existing studies have not yet elucidated the quantitative relationships between this effect and workability, hydration process, and compressive strength of alkali-activated composite cement-based cementitious materials, resulting in difficulties in achieving precise adaptive regulation of alkali-activated composite cement-based cementitious materials for multiple application scenarios. This study addresses this gap by employing TS and FA, a representative industrial byproduct, as composite precursors. Alkali-activated composite cementitious materials (AACC) were synthesized through partial cement replacement combined with alkali activation technology. Utilizing a two-variable experimental design, the investigation systematically examines the multidimensional impact patterns of TS-FA composite content and AE on critical performance parameters: workability (flowability and setting time), hydration processes (chemically bound water content and pore solution pH), and compressive strength. The research outcomes provide theoretical foundations and empirical data, which can advance the effective utilization of industrial solid wastes.

## 2. Results and Discussion

### 2.1. Effects of Sludge Dosage and Alkali Equivalent on Workability

#### 2.1.1. Influence of Sludge Dosage on Flowability

**Figure 1:**
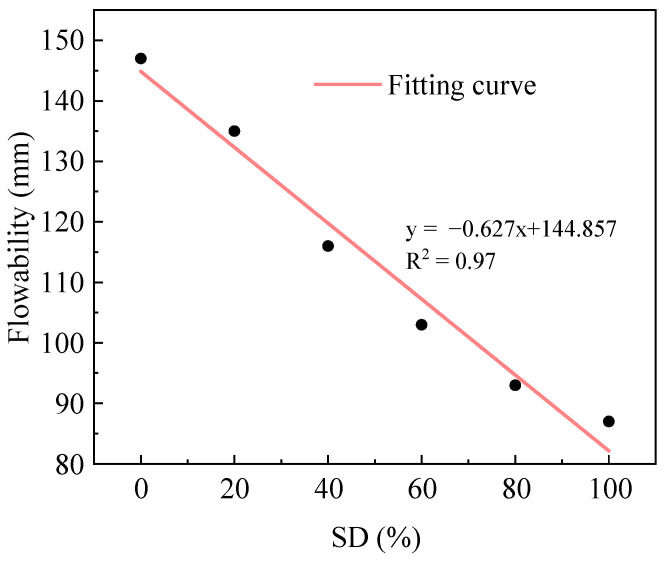
Flowability of AACC under different SD.

Figure 1 shows the change in flowability of fresh AACC under different SD. Through linear regression analysis of the relationship between sludge content and fluidity, the fitting results showed a coefficient of determination R^2^ of 0.98, a mean squared error (MSE) of 11.72 mm^2^, and a mean absolute error (MAE) of 3.23 mm; despite certain errors, the linear model still adequately described the relationship between the two. As illustrated, the flowability exhibits a decreasing trend with increasing SD: a slow decline of 8.2% at 0–20% SD, a slightly accelerated reduction of 14.1% at 20–40%, followed by progressively diminishing decreases of 11.2%, 9.7%, and 6.5% at 40–100%, indicating gradual stabilization of paste flowability at higher sludge incorporation levels. Mechanically processed TS exhibits reduced particle size, increased specific surface area, and porous surface morphology, characteristics that enhance its water absorption capacity [32]. Additionally, the sludge contains abundant organic matter, lipids, and surface hydrophilic functional groups, imparting both hygroscopic and viscous properties [33]. Compared to the flowability of cement paste, the flowability of M0 increased by 8.9%, while M2 showed comparable flowability to the cement paste. However, M4, M6, M8, and M10 exhibited reductions of 14.1%, 23.7%, 31.1%, and 35.6%, respectively. The enhanced flowability observed with FA incorporation stems from its spherical microstructure, which generates a “ball bearing effect” within the cementitious system, significantly improving flowability [34]. At lower sludge incorporation levels (0–20%), this ball bearing effect dominates, maintaining stable flowability without significant decline. However, as SD increases beyond 20%, the material’s high water absorption capacity and inherent viscosity progressively counteract this beneficial effect, leading to marked flowability reduction.

#### 2.1.2. Impact of Sludge Dosage on Setting Behavior

**Figure 2:**
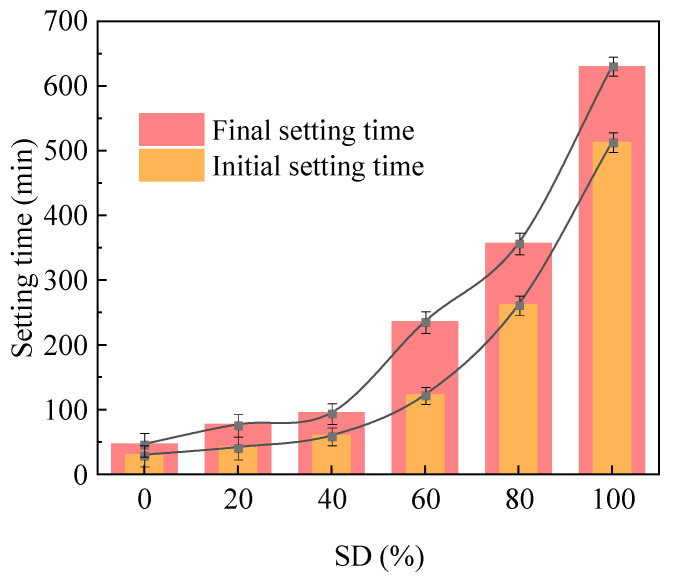
Setting time of AACC under different SD.

Figure 2 illustrates the influence of SD on the setting time of AACC. The results demonstrate an exponential growth trend in the setting time of fresh AACC with increasing SD. The increments in initial setting time across different dosage groups were 40%, 42.9%, 105%, 113%, and 95.8%, while the corresponding increments in final setting time were 63.8%, 23.4%, 148%, 51.2%, and 76.5%. At lower SD, the setting process remained rapid, with initial setting times under 1 h and final setting times within 2 h. However, when SD exceeded 40%, the setting time increased significantly, reaching initial setting times over 2 h and final setting times exceeding 3 h. The low pozzolanic activity of FA primarily results from its predominant silico aluminous glass phase composition, characterized by a low calcium silicon ratio and highly polymerized [SiO_4_]^2−^ tetrahedral structures [35]. However, under alkali activation, rapid cleavage of Si-O and Al-O bonds in both cement and FA glass phases significantly enhance the dissolution rate of reactive components and accelerates polymerization reactions, thereby shortening the paste’s setting time. With increasing TS incorporation, the organic matter content in the alkali-activated composite system rises. At lower dosages, C-S-H gel, the hydration products generated from FA and cement through alkali activation effectively encapsulate organic substances. Although organic matter exhibits certain erosive effects on hydration products, its influence on setting time remains negligible compared to the substantial production of hydration products [36]. At higher SD, the combined effects of the material’s elevated water absorption capacity and increased organic content led to dual mechanisms: partial absorption of hydration water by sludge particles and decomposition of hydration products by organic compounds, consequently prolonging the setting time proportionally with SD.

#### 2.1.3. Influence of Alkali Equivalent on Flowability

**Figure 3:**
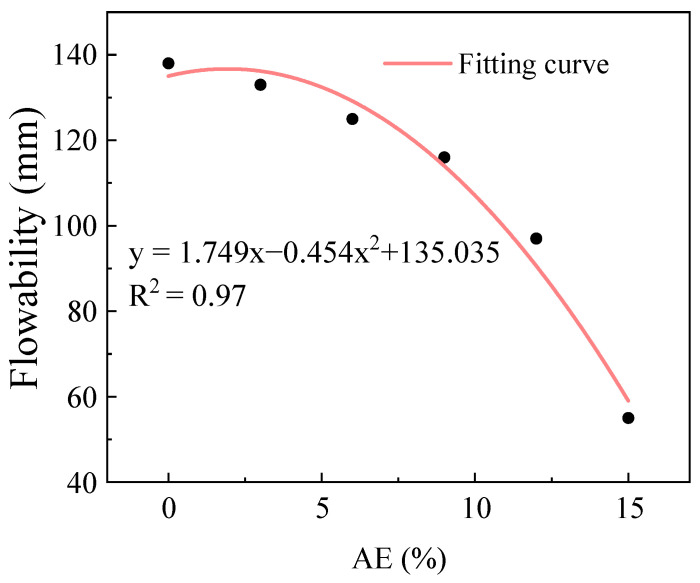
Flowability of AACC under different AE.

Figure 3 illustrates the variation in flowability of fresh AACC under different AE. Based on the fitting results of alkali equivalent and fluidity, the coefficient of determination R^2^ was 0.98, the MSE was 16.31 mm^2^, and the MAE was 3.8 mm; the results adequately described the relationship between the two. The results show an overall declining trend in flowability with increasing AE, accompanied by an amplified reduction rate. When the AE ranges from 0% to 9%, the impact on the flowability is minimal, with reductions ranging between 3.6% and 7.2%. However, when the AE exceeds 9%, the flowability decreases sharply—a 16.3% drop at 12% AE compared to 9%, and a further 43.3% reduction at 15% AE, resulting in a flowability of only 55 mm, indicating near complete loss of flowability. This phenomenon occurs because the modification of powder particles by alkaline solutions induces charge imbalance, leading to the formation of flocculent structures through van der Waals forces, thermal motion, and particle collisions, thereby reducing flowability [37]. Additionally, flowability is closely related to plastic viscosity, which reflects the internal resistance to fluid flow. Under constant external forces, higher plastic viscosity results in greater difficulty in paste flow. In the AACC experiments, where the system was subjected only to relatively constant gravitational force, the incorporation of alkaline solutions significantly increased the plastic viscosity of the paste. Consequently, the fresh mortar exhibited increasingly restricted flow under gravity, resulting in pronounced flowability loss [38,39].

#### 2.1.4. Impact of Alkali Equivalent on Setting Behavior

**Figure 4:**
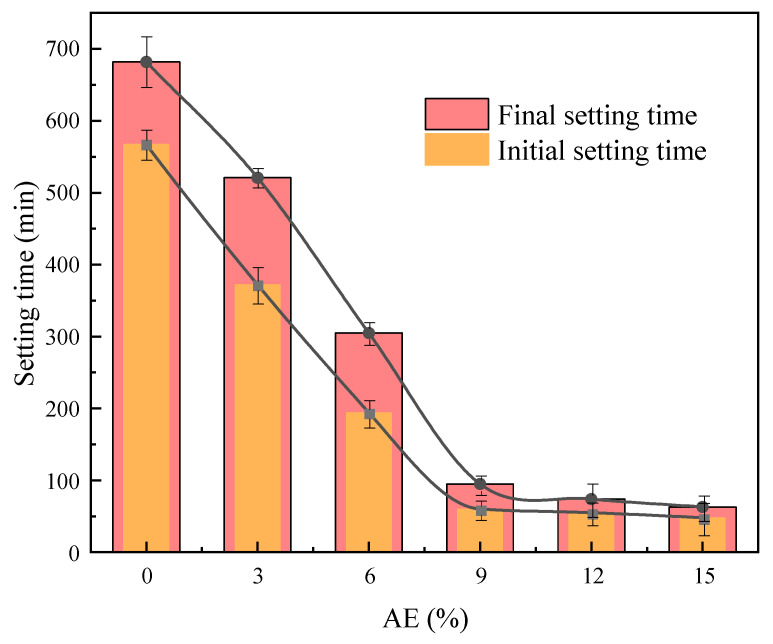
Setting time of AACC under different AE.

Figure 4 shows the variation in setting time of fresh AACC under different AE. For the control group A0, the initial setting time exceeds 9 h and the final setting time exceeds 11 h, indicating prolonged setting characteristics. When the AE reaches 9%, the initial setting time of the alkali-activated composite system shortens to 1 h, and the final setting time reduces to 1 h and 35 min. This is primarily due to the high organic content in TS, where acidic components react with hydration products to form soluble salts, hindering the bonding between hydration products and sludge particles. The alkali activator neutralizes acidic groups in the sludge, increases alkalinity in the paste, effectively degrades organic matter, and promotes the release of SiO_2_ and Al_2_O_3_ from layered silicate minerals in the sludge, thereby accelerating the reaction rate and shortening the setting time. When the AE ranges from 0% to 9%, the setting time of the alkali-activated composite system decreases significantly by 23.6%, 41.5%, and 68.9%, respectively. When the AE exceeds 9%, the setting time stabilizes with reductions between 14% and 23%, exhibiting an overall exponential decline trend. These findings align with existing research results [40,41,42].

### 2.2. Hydration Process Evolution Under Composite Admixture Variations

#### 2.2.1. Effect of Sludge Dosage on Chemically Bound Water

**Figure 5:**
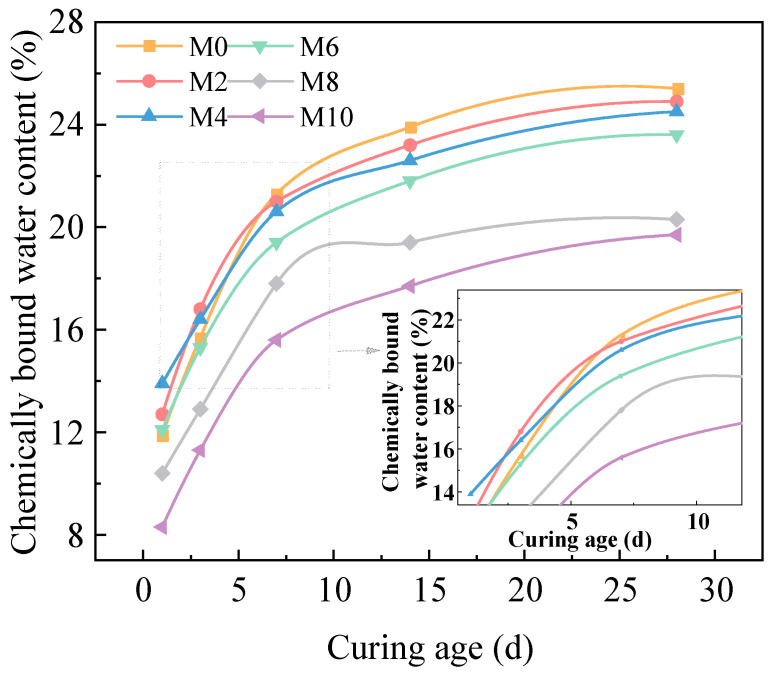
Chemically bound water content of AACC under different SD.

Figure 5 shows the variation curves of chemically bound water content in hardened AACC with different SD under ambient curing conditions as the curing age increases. The results reveal that in the early hydration stage, the chemically bound water content initially increases and then decreases with increasing SD. At 7 d of curing, the chemically bound water content gradually declines with increasing SD, and this trend remains consistent in later stages. By 28 d, the chemically bound water content stabilizes at 23–26% for M0, M2, M4, and M6. Compared to M6, M8 and M10 exhibit reductions of 8.2% and 16.5%, respectively. This behavior is attributed to the high proportion of silicon phases in FA, where [SiO_4_]^2−^ forms a continuous three dimensional network structure within particles, resulting in low pozzolanic activity during early stages. TS contains CaO, which reacts with water to generate Ca(OH)_2_, enhancing alkalinity and promoting silicon–aluminum reactions [43]. Thus, moderate sludge incorporation increases chemically bound water content in early hydration. In later stages, FA participates in hydration reactions, increasing geopolymer gel content in low sludge groups (≤60%). However, when SD exceeds 60%, excessive organic matter consumption of hydration products maintains chemically bound water at lower levels. Notably, the chemically bound water content of pure cement specimens at various ages resembles that of group M8, indicating that alkali activation significantly enhances hydration reactions in low activity cementitious systems containing FA and TS.

#### 2.2.2. pH Dynamics in Pore Solution with Sludge Dosage

**Figure 6:**
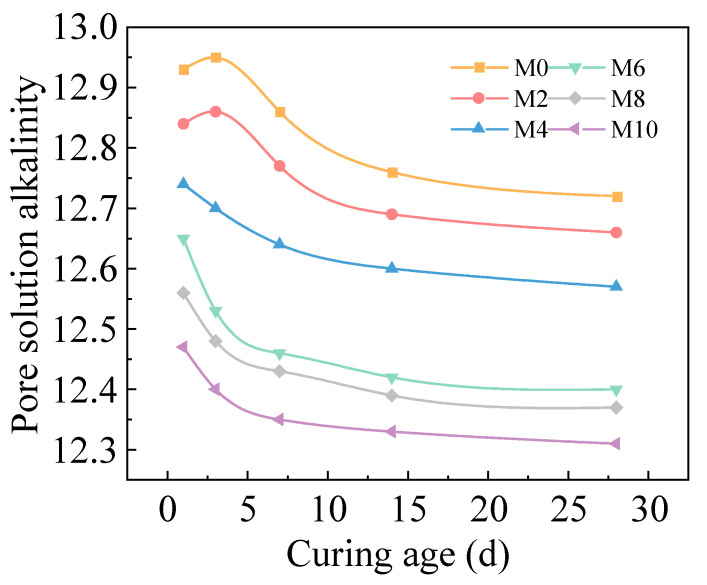
Pore solution alkalinity of AACC under different SD.

Figure 6 illustrates the temporal evolution of pore solution alkalinity in hardened AACC with different SD during 28 d of hydration. The results demonstrate a continuous decrease in pore solution alkalinity with increasing TS content. At 1 d, the pore solution pH of group M10 measured 12.47, declining to 12.31 by 28 d. In pure cement specimens, the OH^−^ concentration in pore solution rises rapidly during early hydration, with pH values increasing sharply within 14 d before stabilizing around 12.87. For groups M0 and M2, alkali-activated dissolution rapidly releases monovalent alkali ions into the pore solution. Although partially adsorbed by C-S-H gel, the pore solution alkalinity shows no significant reduction during initial stages, even exhibiting slight increases before stabilizing. Following this transient equilibrium, pozzolanic reactions between FA and Ca(OH)_2_ generate substantial low calcium–silicon ratio C-S-H gel, which exhibits stronger alkali fixation capacity compared to high Ca/Si C-S-H gel from cement hydration, leading to progressive alkalinity reduction until stabilization after 28 d. In M6, M8, and M10, acidic organic components react with Ca(OH)_2_ during hydration to form soluble salts, thereby causing a more rapid decline in pore solution alkalinity.

#### 2.2.3. Effect of Alkali Equivalent on Chemically Bound Water

**Figure 7:**
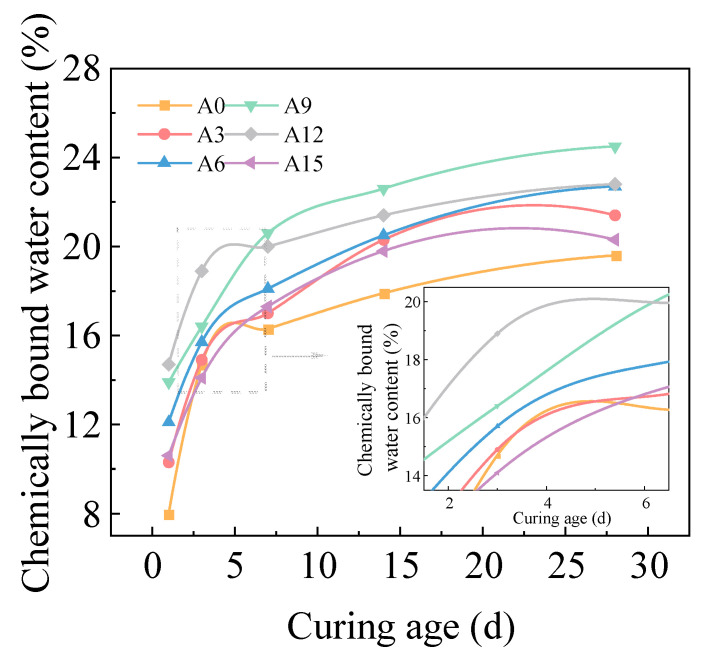
Chemically bound water content of AACC under different AE.

Figure 7 displays the variation curves of chemically bound water content in hardened AACC under different AE as curing age progresses. The results reveal that chemically bound water content initially increases and then decreases with rising AE. At 1d of curing, the A12 group exhibits the highest chemically bound water content (14.7%), representing an 83.8% increase compared to the control group. By 28 d, the A9 group achieves the maximum chemically bound water content (24.5%), showing a 25% enhancement over the control. This phenomenon arises from the inhibitory effects of oils and organic substances in TS on hydration reactions, leading to incomplete cement hydration. During the initial hydration phase, FA primarily acts as a physical filler, with cement remaining the dominant participant in hydration reactions, resulting in reduced formation of hydration products and consequently lower chemically bound water content in group A0. The incorporation of alkali activators elevates [SiO_4_]^2−^ and OH^−^ ion concentrations in the alkali-activated composite system, facilitating the disintegration and dissolution of Si and Al phases within the vitreous components of cementitious materials [44], thereby enhancing geopolymer gel production and hydration degree. However, excessive AE accelerate surface hydration product deposition on cement particles, creating diffusion barriers that impede complete internal hydration and reduce chemically bound water content. These observations align with microstructural analyses where optimal AE promote hydration product formation, while excessive levels induce porous structures. Throughout the curing period, chemically bound water content reaches its minimum at 1d but demonstrates the most rapid growth rate during this phase. All groups exhibit fast hydration kinetics before 7 d, followed by gradual deceleration after 7 d, with hydration stabilization occurring beyond 14 d. The negligible difference between 14 d and 28 d values indicates that the alkali-activated composite system attains substantial hydration completion by 14 d.

#### 2.2.4. pH Dynamics in Pore Solution with Alkali Equivalent

**Figure 8:**
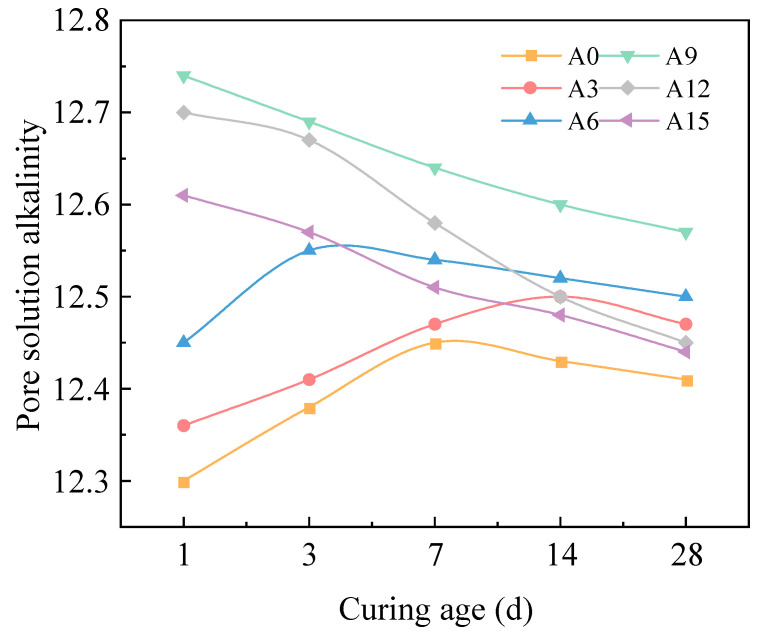
Pore solution alkalinity of AACC under different AE.

Figure 8 illustrates the variation curves of pore solution alkalinity in hardened AACC under different AE across curing ages. The results demonstrate that pore solution alkalinity initially increases and then decreases with rising AE at all tested ages. At 1d of curing, the control group A0 exhibits the lowest pore solution alkalinity (pH 12.3), while the alkali-activated composite system with 9% AE achieves the maximum alkalinity (pH 12.74). The disparity in pore solution alkalinity among groups gradually diminishes with prolonged curing, stabilizing within pH 12.4–12.6 at 28 d. This phenomenon is attributed to the inherent low pH (≈7.8) of TS due to organic matter content, which partially neutralizes alkalinity when incorporated at 8% (by mass of total cementitious materials) into the alkali-activated composite system, thereby reducing pore solution alkalinity to some extent. Additionally, acidic components in organic matter react with hydration derived Ca(OH)_2_, reducing alkalinity in the control group where pore solution alkalinity primarily originates from cement hydration products (Ca(OH)_2_) and alkali metal ions. Alkali activator incorporation intensifies cementitious material hydration, generating abundant OH^−^ and releasing immobilized alkali ions (K^+^, Na^+^) into pore solutions, thereby rapidly elevating alkalinity [45]. However, excessive AE (>9%) accelerate surface hydration, forming dense product layers around cement particles that inhibit internal hydration, ultimately reducing Ca(OH)_2_ and alkali ion generation, leading to alkalinity decline.

Throughout the curing period, the pore solution alkalinity of groups A0, A3, and A6 initially increases rapidly and then decreases gradually with prolonged age, whereas groups A9, A12, and A15 exhibit a slow decline in pore solution alkalinity over time. This occurs because geopolymer gel encapsulates organic matter in the sludge and neutralizes its acidity, with progressive hydration reactions enhancing pore solution alkalinity as curing progresses. Moderate alkali activator addition promotes fuller hydration of cementitious materials, generating additional OH^−^ and alkali metal ions that further elevate pore solution alkalinity. In later stages, the enhanced activation of FA and TS facilitates reactions with Ca(OH)_2_, producing substantial low calcium–silicon ratio C-S-H gel. These gels exhibit alkali fixation capacity, reducing alkali metal content and consequently lowering pore solution alkalinity [46]. At higher AE, accelerated hydration rates intensify the polycondensation of silicon–aluminum monomers, increasing OH^−^ consumption and decreasing residual alkali content, thereby reducing pore solution alkalinity.

### 2.3. Variation Rules of Compressive Strength

#### 2.3.1. Effect of Sludge Dosage on Compressive Strength

**Figure 9:**
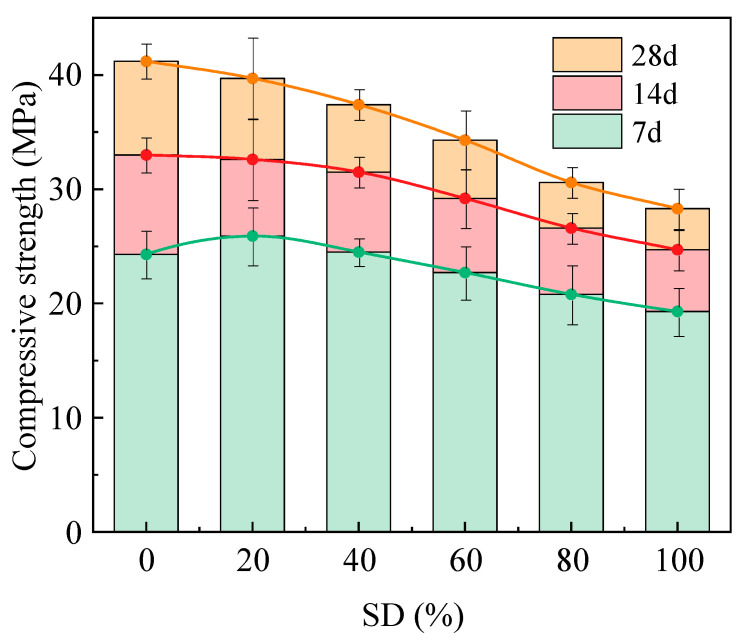
Compressive strength of AACC under different SD.

Figure 9 illustrates the variation in compressive strength of AACC specimens with different SD during early and later curing stages. The results indicate that the compressive strength of alkali-activated specimens continuously increased with prolonged curing ages across all SD. From 3 d to 7 d, the strength growth rates for groups M0, M2, M4, M6, M8, and M10 were 35.8%, 29.1%, 28.6%, 28.6%, 27.9%, and 28%, respectively. From 7 d to 28 d, the corresponding growth rates decreased to 24.8%, 21.8%, 18.7%, 17.5%, 15%, and 14.6%, with the growth rates exhibiting an overall declining trend as SD increased. This demonstrates the detrimental effects of TS on AACC hydration, where higher SD progressively impedes hydration kinetics, evidenced by reduced strength increments between curing ages. Notably, at 3 d, the compressive strength initially increased then decreased with SD: groups M2 and M4 exhibited higher strengths than M0. This is attributed to the excessive water to binder ratio in M0, where limited early hydration water left abundant free water in the matrix. Moderate sludge incorporation absorbed excess water, reducing free water content and enhancing early densification. However, excessive sludge addition caused excessive water loss, lowering chemically bound water content and hydration degree. With prolonged curing, free water in M0 gradually converted into chemically bound water, while hydration products filled internal pores, achieving maximum compressive strengths of 33 MPa (7 d) and 41.2 MPa (28 d). This phenomenon primarily stems from the predominance of organic matter in TS, with only a minor proportion of active components (CaO and SiO_2_) participating in hydration reactions [47]. Given a fixed total content of FA and TS, increased sludge incorporation elevates organic matter content while reducing FA proportion. This dual effect weakens interparticle cohesion in cementitious systems, diminishes active aluminosilicate availability, and decreases hydration products formation. Consequently, the increased incorporation of TS leads to diminished compressive strength in solidified matrices.

#### 2.3.2. Effect of Alkali Equivalent on Compressive Strength

**Figure 10:**
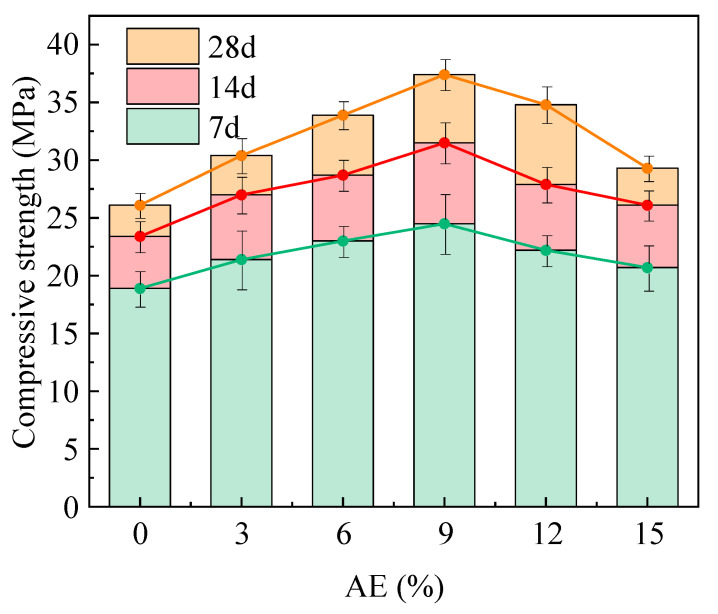
Compressive strength of AACC under different AE.

The AE significantly influences both early mechanical properties and later strength development of AACC, as shown in Figure 10. The compressive strength of alkali-activated specimens with different AE continuously increased with prolonged curing ages. From 3 d to 7 d curing ages, the strength growth rates for groups A0, A3, A6, A9, A12, and A15 were 23.8%, 26.1%, 24.8%, 28.6%, 25.7%, and 26.1%, respectively. From 7 d to 28 d curing ages, the corresponding growth rates decreased to 11.5%, 12.6%, 18.1%, 18.7%, 24.7%, and 12.3%, exhibiting an initial increase followed by a decline trend with higher AE. Notably, all alkali-activated groups demonstrated superior strength growth trends compared to the control group (A0) across curing ages, indicating that the alkali activator consistently promoted hydration reactions in the composite cementitious material and mitigated the adverse effects of TS participation in hydration throughout the entire curing period. Furthermore, as the AE increases, the compressive strength of alkali-activated specimens at all ages exhibits a trend of initial increase followed by decrease, reaching maximum values at 9% AE with 3 d, 7 d, and 28 d compressive strengths of 24.5, 31.5, and 37.4 MPa, respectively, representing 29.6%, 34.6%, and 43.3% improvements compared to the control group. At 12% AE, the compressive strengths at 3 d, 7 d, and 28 d decreased by 9.4%, 11.4%, and 7%, respectively, compared to the 9% AE group, while at 15% AE, the reductions reached 15.5%, 17.1%, and 21.7%, respectively. This demonstrates the feasibility of incorporating alkali activators in TS/FA composite cementitious materials, where appropriate AE significantly improves the hydration environment of cement mineral components, accelerates hydration processes, and enhances both early and long-term strength development of AACC. However, under high alkalinity conditions, the primary hydration product C-S-H gel becomes unstable and more susceptible to damage during drying, leading to volumetric instability and shrinkage cracks that compromise strength development.

### 2.4. Hydration–Strength Relationships of AACC

#### 2.4.1. Hydration–Strength Relationships Under Sludge Dosage Gradients

**Figure 11:**
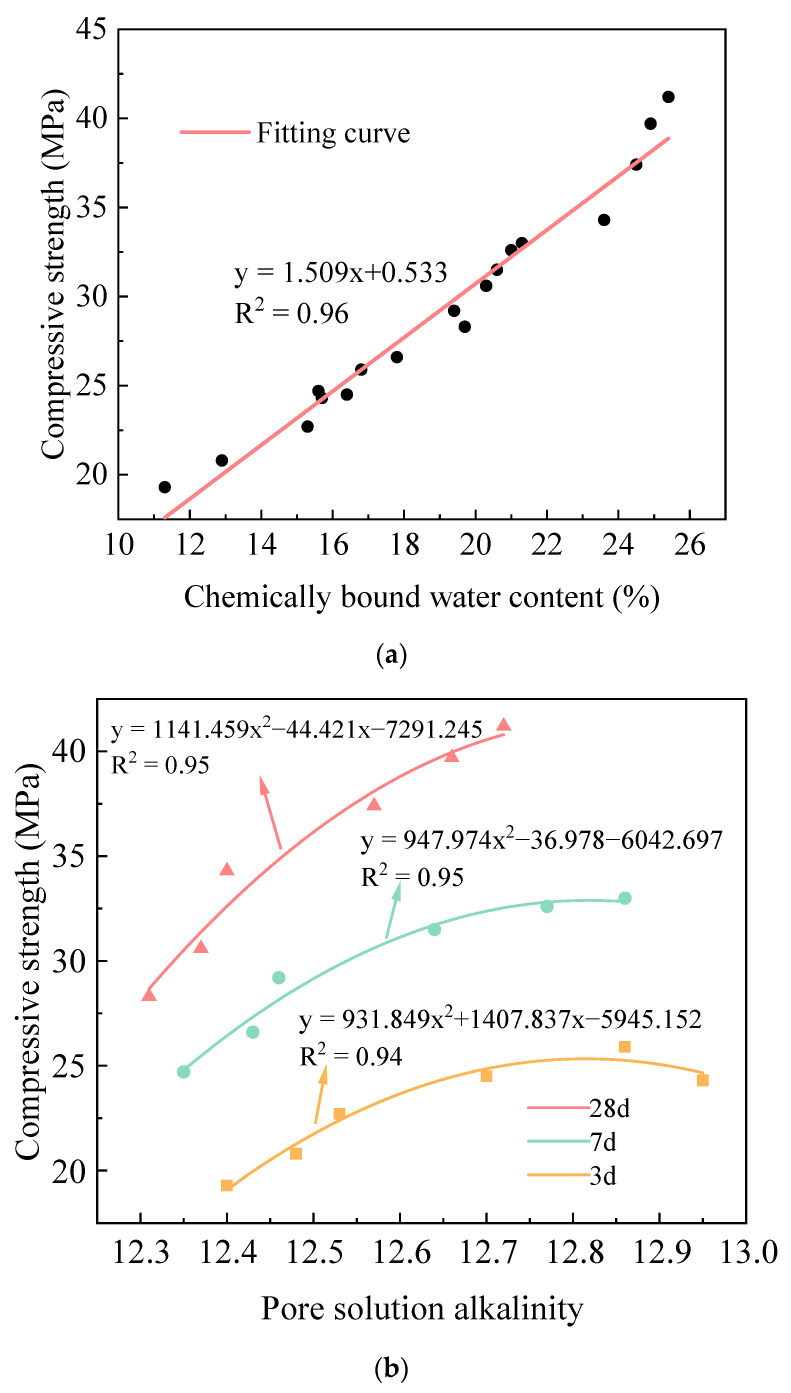
Relationship between AACC compressive strength and hydration process under different SD. (**a**) Relationship between compressive strength and chemically bound water content. (**b**) Relationship between compressive strength and pore solution alkalinity.

Chemically bound water content and pore solution alkalinity, as two critical indicators for characterizing the hydration degree of cementitious materials, exhibit an intrinsic connection with their strength development. The hydration process of AACC, influenced by alkali activators and cementitious material composition, involves complex hydration reactions and strength evolution. To quantitatively analyze the relationship between AACC compressive strength and chemically bound water content/pore solution alkalinity, compressive strength data from 3 d, 7 d, and 28 d curing ages under different SD were systematically correlated with these two parameters, establishing mathematical models as shown in Figure 11. In Figure 11a, the coefficient of determination R^2^ for the fitting results between chemically bound water and compressive strength was 0.96, the MSE was 1.24 MPa^2^, and the MAE was 0.87 MPa; the results adequately described the relationship between the two. Figure 11a reveals that AACC compressive strengths range between 19–42 MPa with chemically bound water content spanning 15% to 26%. The compressive strength demonstrates a linear increasing trend with chemically bound water content, achieving a determination coefficient R^2^ value of 0.97, indicating strong linear correlation between these parameters across SD and curing ages. As shown in Figure 11b, the pore solution alkalinity of AACC ranges from 12.3 to 12.95, with data points corresponding to compressive strength exhibiting distinct stratification based on curing ages. The compressive strength is divided into three intervals, each demonstrating a univariate quadratic relationship with pore solution alkalinity, where strength increases with rising alkalinity, achieving coefficients of determination (R^2^) above 0.94; the MSE did not exceed 0.18 MPa^2^, and the MAE did not exceed 0.7 MPa. Additionally, as curing age progresses, the pore solution alkalinity decreases across all SD, shifting leftward overall, while compressive strength increases, indicating that curing age significantly influences compressive strength under varying sludge incorporation levels.

**Figure 12:**
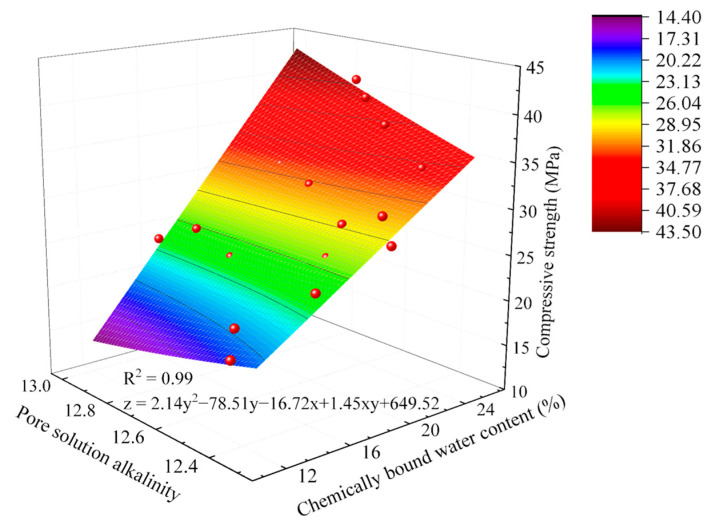
Multivariate function model between AACC compressive strength and hydration process under different SD.

As revealed by the fitting results in Figure 12, compressive strength exhibits a linear relationship with chemically bound water content, while demonstrating univariate quadratic relationships with pore solution alkalinity across different curing ages. To establish a more comprehensive and precise mathematical model, it is essential to concurrently consider the influences of both chemically bound water content and pore solution alkalinity. Building upon the aforementioned analytical findings, a multifactorial strength model was developed, as presented in Equation (1).

(1) $$f_{c}=aA_{n}^{2}+bA_{n}+cW_{n}+dA_{n}W_{n}+e$$

where *f_c_* is the compressive strength of AACC (MPa); *A_n_* is the pore solution alkalinity; *W_n_* is the chemically bound water content; and *a*, *b*, *c*, *d* and *e* are regression coefficients.

Based on Equation (1), a mathematical model between the compressive strength of AACC and hydration degree indicators under different SD from 3 d to 28 d curing ages was established, as shown in Equation (2).

(2) $$f_{c}=2.14A_{n}^{2}-78.51A_{n}-16.72W_{n}+1.45A_{n}W_{n}+649.52$$

As shown in Figure 12, the multivariate function model for compressive strength achieves an R^2^ value of 0.99. After F-test analysis revealed a significance probability (*p*-value) far below 0.05, the model demonstrates a highly significant relationship between compressive strength and chemically bound water content/pore solution alkalinity under this analytical framework. Compared to the aforementioned two-dimensional models, this model exhibits superior representativeness and applicability.

#### 2.4.2. Hydration–Strength Relationships Under Alkali Equivalent Gradients

**Figure 13:**
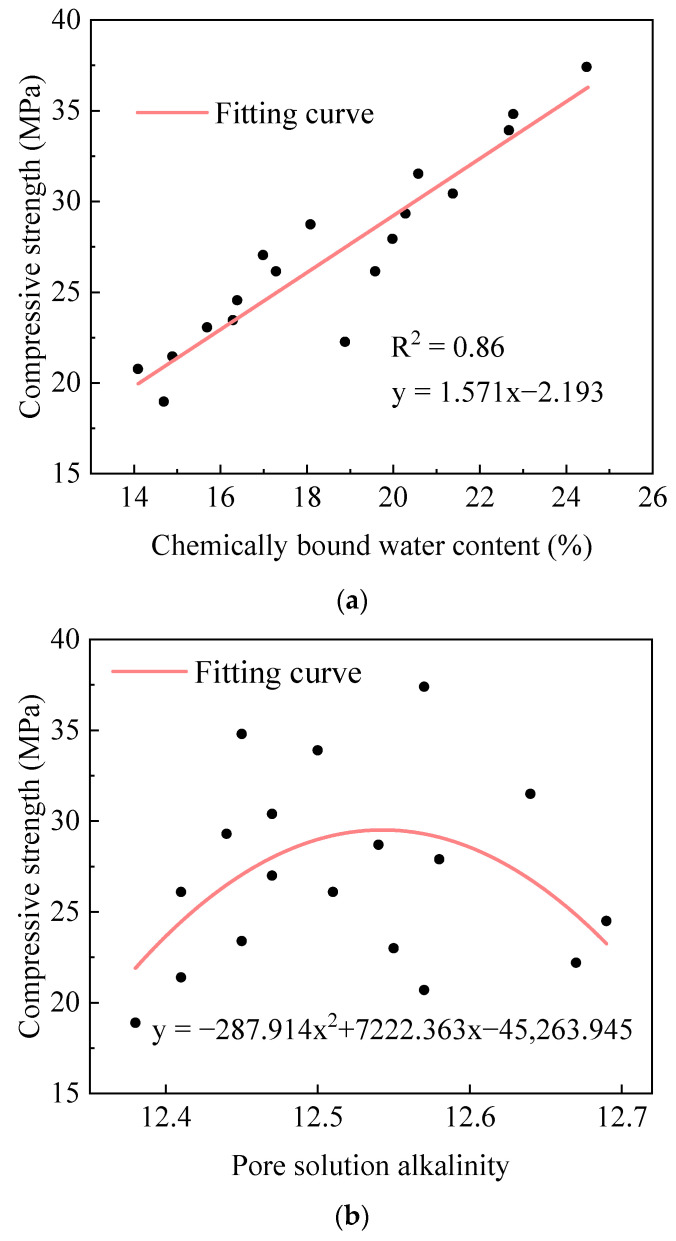
Relationship between AACC compressive strength and hydration process under different AE. (**a**) Relationship between compressive strength and chemically bound water content. (**b**) Relationship between compressive strength and pore solution alkalinity.

By integrating the compressive strength data from 3 d, 7 d, and 28 d curing ages under different AE with the two indicators (chemically bound water content and pore solution alkalinity), mathematical models between compressive strength and both parameters were established, as shown in Figure 13. In Figure 13a, the coefficient of determination R^2^ for the fitting results between chemically bound water and compressive strength was 0.86, the MSE was 3.39 MPa^2^, and the MAE was 1.39 MPa; the results adequately described the relationship between the two. The compressive strength of AACC ranges between 18 and 38 MPa, while the chemically bound water content varies from 14% to 25%, with compressive strength exhibiting a linear increasing trend as chemically bound water content rises, achieving a coefficient of determination R^2^ of 0.86, indicating a high degree of linear correlation between chemically bound water content and compressive strength in AACC. As shown in Figure 13b, the pore solution alkalinity of AACC ranges from 12.35 to 12.7, where the data points corresponding to compressive strength exhibit significant scatter with weak correlation, approximately following a univariate quadratic functional relationship, yielding a low R^2^ value of 0.21; the MSE was 19.86 MPa^2^, and the MAE was 3.73 MPa.

**Figure 14:**
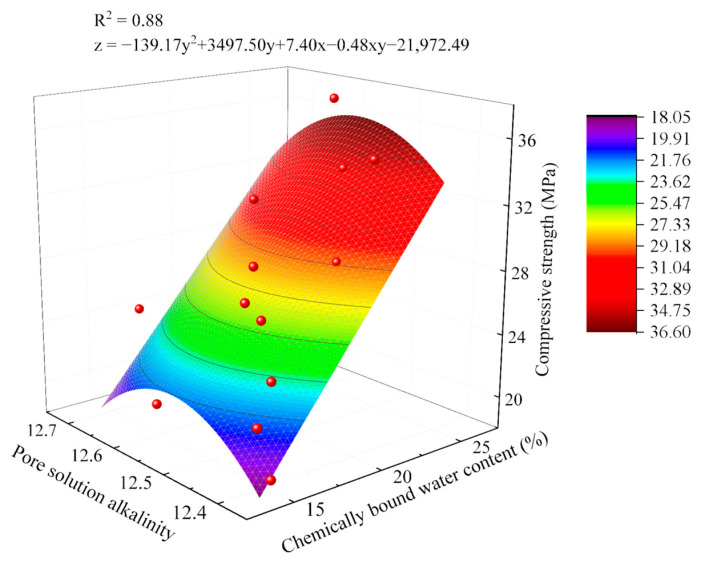
Multivariate function model between AACC compressive strength and hydration process under different AE.

As shown in the fitting results in Figure 14, the correlation between compressive strength and chemically bound water content is significant, while the correlation with pore solution alkalinity is less pronounced but generally follows a quadratic functional trend of initial increase followed by decrease. Based on Equation (1), the relationship between AACC compressive strength and chemically bound water content/pore solution alkalinity under varying AE was derived through multivariate regression, and a multifactorial function model was established, as presented in Equation (3).

(3) $$f_{c}=-125.34A_{n}^{2}+3149.65A_{n}+6.58W_{n}-0.41A_{n}W_{n}-19786.23$$

As shown in Figure 14, the R^2^ values of most compressive strength models reach 0.873, and F-test analysis revealed significance probabilities (*p*-values) all below 0.05. Consequently, this mathematical model demonstrates good agreement with experimental results and can accurately describe the quantitative relationship between AACC compressive strength and hydration degree indicators under varying AE across different curing ages.

## 3. Conclusions

The flowability of AACC exhibits a declining trend with increasing SD and AE, demonstrating superior flowability when FA content is high and AE is low. Both the initial and final setting times of the paste follow exponential upward trends with increasing SD and exponential downward trends with rising AE. Increasing sludge incorporation or reducing AE can effectively prolong the setting time, providing practical guidance for engineering applications.

Increasing the SD in AACC elevates organic matter content and promotes the formation of low calcium-to-silica ratio C-S-H gel, leading to reduced chemically bound water content and pore solution alkalinity, thereby exerting an inhibitory effect on cement hydration reactions. Appropriately increasing the AE can facilitate the disintegration and dissolution of Si- and Al-phases in the AACC glassy matrix, generating additional C-S-H gel while enhancing both chemically bound water content and pore solution alkalinity.

Increasing SD moderately enhances early compressive strength of AACC but impedes mid to late strength development. Reduced SD elevates chemically bound water content, with compressive strength demonstrating a linear growth trend (maximum value: 41.2 MPa). Across curing ages, pore solution alkalinity and compressive strength exhibit a univariate quadratic functional relationship, where strength increases with rising alkalinity. Elevated AE induce an initial increase followed by a decrease in compressive strength at all curing ages, peaking at 37.4 MPa with 9% AE. The consistent relationship between chemically bound water content and compressive strength under varying AE and SD confirms that chemically bound water content effectively characterizes strength variations in AACC. 

The adjustability of ACCA’s fluidity, setting time, and compressive strength allows it to be combined with aggregates to produce concretes with diverse functionalities, such as pumped concrete, retarded-setting concrete, and early-strength concrete, demonstrating significant application potential in the field of civil engineering while combining economic efficiency with environmental benefits.

## 4. Materials and Methods

### 4.1. Raw Materials

1. Cement.
  The experimental study employed P.O 42.5 ordinary Portland cement produced by Henan Tianrui Group Co., Ltd (Pingdingshan, China) with a specific surface area of 348.7 m^2^/kg (measured via Blaine method) and a density of 3120 g/m^3^.
2. Composite admixture.
  The composite admixture consisted of TS and FA. The FA, classified as Grade F low calcium FA, was produced by Borun Refractory Materials Co., Ltd (Gongyi, China) exhibiting a specific surface area of 330 m^2^/kg and a density of 2550 kg/m^3^. The raw TS was obtained from Henan Yongsheng Leather Industry Co., Ltd (Zhumadian, China) after concentration and filter pressing. Through laboratory processing including drying, crushing, mechanical grinding, and sieving, TS powder with particle sizes below 0.15 mm was prepared, demonstrating a specific surface area of 310 m^2^/kg and density of 1850 kg/m^3^. The chemical composition of the cementitious materials is presented in Table 1.

3. Alkali activator.
  The alkali activator used in the experiment was formulated through composite blending of sodium hydroxide (NaOH) and sodium silicate solution (Na_2_SiO_3_). The sodium hydroxide, with a purity of 95%, primarily served to adjust the modulus and alkalinity of the sodium silicate. The industrial grade sodium silicate solution (water glass) was supplied by Yourui Refractory Materials Co., Ltd (Jiaxing, China) exhibiting a transparent viscous state.

### 4.2. Mix Proportion Design and Preparation Process

In this experiment, the alkali-activated composite cementitious material was formulated with a water to binder ratio of 0.4. Six distinct AACC formulations were prepared by varying the blending ratios of TS and FA, with the alkali activator modulus adjusted to 1.5. The combined proportion of “TS + FA” was fixed at 20% of the total cementitious materials, while the relative contribution of TS within this 20% blend was systematically altered to investigate its impact on the performance of cement–FA pastes. Specifically, the TS content was set at 0%, 20%, 40%, 60%, 80%, and 100% of the total FA–TS mixture (by mass), as detailed in Table 2. The experimental results revealed that the 40% TS formulation (designated as M40) exhibited the highest compressive strength. Consequently, this optimal blend was selected as the baseline for further investigation into the effects of AE variations. The alkali equivalent (AE)—defined as the mass percentage of Na_2_O in the alkali activator relative to the total mass of FA and TS—was systematically adjusted to 0%, 3%, 6%, 9%, 12%, and 15%, as outlined in Table 3.

**Figure 15:**
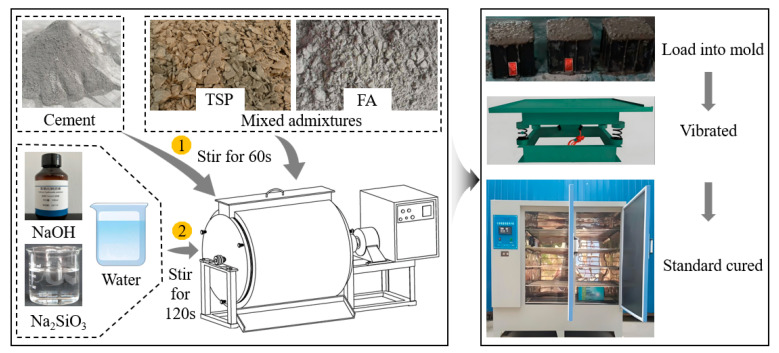
AACC Preparation flow chart.

The preparation process is shown in Figure 15.

### 4.3. Test Method

1. The compressive strength test of AACC refers to Test Method for Cement Mortar Strength (ISO Method) (GB/T17671-2021) [48]. Cubic specimens with dimensions of 40 × 40 × 40 mm are prepared, with three samples per group. An electro-hydraulic servo pressure testing machine is used to measure the compressive strength of specimens cured for 7 d and 28 d.
2. The test selected flowability as the indicator for flowability evaluation, following the cement paste flowability test method specified in Methods for testing uniformity of concrete admixtures (GB/T8077-2023) [49]. Additionally, the setting time of fresh alkali-activated composite cementitious paste was determined according to Test Methods for Water Requirement of Normal Consistency, Setting Time, and Soundness of Cement (GB/T1346-2011) [50].
3. The chemically bound water content is measured using the loss on ignition method as follows: hardened paste specimens cured to the specified age are immersed in anhydrous ethanol to terminate hydration. The specimens are then manually crushed, ground into powder, and sieved through a 0.08 mm square mesh sieve. The sieved powder is dried in an oven at 75 °C until a constant mass is achieved. The dried powder mass (*m*_1_) is weighed using an analytical balance. The dried powder is heated in a muffle furnace to 1050 °C for 3 h. After cooling to ambient temperature, the mass is reweighed (*m*_2_). The chemically bound water content *W_n_* of the sample is calculated as follows:
  (4) $$W_{n}=\frac{\left(m_{1}-m_{2}\right)}{m_{1}-L}\cdot \frac{1}{1-L}$$
  (5) $$L=\beta_{C}L_{C}+\beta_{TS}L_{TS}+\beta_{FA}L_{FA}$$
  In the formula, *L_C_*, *L_TS_*, and *L_FA_* represent the loss on ignition of cement, TS, and FA, respectively; *β* denotes the mass fraction of cement and supplementary cementitious materials in the composite cementitious system.
4. The alkalinity of pore solution is tested using the extraction leaching method with the following specific steps: after terminating the hydration of hardened paste specimens with different mix proportions at various ages, grind them into powder and sieve through a 0.08 mm square mesh sieve; weigh 3 g of the sieved powder and place it in a beaker; add 30 g of distilled water and stir with a magnetic stirrer for 0.5 h (at 200 rpm); let it stand for 12 h, then test the pH value of the supernatant using a Leici PHS-25 pH meter.

## Figures and Tables

**Table 1:** Chemical composition of cementitious materials (wt.%).

Ingredient	CaO	SiO_2_	Al_2_O_3_	Fe_2_O_3_	MgO	Na_2_O	K_2_O	SO_3_	TiO_2_	P_2_O_5_	Cr_2_O_3_
Cement	58.4	18.8	5.04	3.18	5.58	2.08	1.51	4.82	0.248	-	-
FA	5.6	45.1	24.2	11.85	0.87	1.02	-	2.1	0.82	0.85	-
TS	33.5	6.72	3.04	17.5	2.84	5.27	0.53	20.5	0.618	2.59	5.49

**Table 2:** TS mixture ratio (kg/m^3^).

Groups	Proportion of TS	Cementitious Materials			Sodium Silicate	NaOH	Water
		Cement	TS	FA			
M0	0%	1333.6	0	333.40	159.71	21.12	666.8
M20	20%	1333.6	66.68	266.72	159.71	21.12	666.8
M40	40%	1333.6	133.36	200.04	159.71	21.12	666.8
M60	60%	1333.6	200.04	133.36	159.71	21.12	666.8
M80	80%	1333.6	266.72	66.68	159.71	21.12	666.8
M100	100%	1333.6	333.40	0	159.71	21.12	666.8

**Table 3:** AE mixture ratio (kg/m^3^).

Groups	AE	Cementitious Materials			Sodium Silicate	NaOH	Water
		Cement	TS	FA			
A0	0%	1333.6	133.36	200.04	0	0	666.8
A3	3%	1333.6	133.36	200.04	58.56	6.45	629.23
A6	6%	1333.6	133.36	200.04	117.12	12.91	591.66
A9	9%	1333.6	133.36	200.04	175.68	19.36	554.08
A12	12%	1333.6	133.36	200.04	234.23	25.81	516.52
A15	15%	1333.6	133.36	200.04	292.80	32.26	478.94

## Data Availability

The original contributions presented in the study are included in the article, further inquiries can be directed to the corresponding author.
